# First RNA-seq approach to study fruit set and parthenocarpy in zucchini (*Cucurbita pepo L.)*

**DOI:** 10.1186/s12870-019-1632-2

**Published:** 2019-02-06

**Authors:** Teresa Pomares-Viciana, Mercedes Del Río-Celestino, Belén Román, Jose Die, Belén Pico, Pedro Gómez

**Affiliations:** 1 Genomics and Biotechnology Department, IFAPA Research Centre La Mojonera, Camino de San Nicolás, 1, 04745 La Mojonera, Almería, Spain; 2 Genomics and Biotechnology Department, IFAPA Research Centre Alameda del Obispo, Avd. Menéndez Pidal s/n, 14004 Córdoba, Spain; 3 Genetics Department, University of Cordoba, Av. de Medina Azahara, 5, 14071 Córdoba, Spain; 4 Institute for the Conservation and Breeding of Agricultural Biodiversity (COMAV-UPV), Universitat Politècnica de València, Camino de Vera s/n, 46022 Valencia, Spain

**Keywords:** *Cucurbita pepo*, Zucchini, Parthenocarpy, Fruit set, Differential gene expression, RNA-seq

## Abstract

**Background:**

Zucchini fruit set can be limited due to unfavourable environmental conditions in off-seasons crops that caused ineffective pollination/fertilization. Parthenocarpy, the natural or artificial fruit development without fertilization, has been recognized as an important trait to avoid this problem, and is related to auxin signalling. Nevertheless, differences found in transcriptome analysis during early fruit development of zucchini suggest that other complementary pathways could regulate fruit formation in parthenocarpic cultivars of this species. The development of next-generation sequencing technologies (NGS) as RNA-sequencing (RNA-seq) opens a new horizon for mapping and quantifying transcriptome to understand the molecular basis of pathways that could regulate parthenocarpy in this species. The aim of the current study was to analyze fruit transcriptome of two cultivars of zucchini, a non-parthenocarpic cultivar and a parthenocarpic cultivar, in an attempt to identify key genes involved in parthenocarpy.

**Results:**

RNA-seq analysis of six libraries (unpollinated, pollinated and auxin treated fruit in a non-parthenocarpic and parthenocarpic cultivar) was performed mapping to a new version of *C. pepo* transcriptome, with a mean of 92% success rate of mapping. In the non-parthenocarpic cultivar, 6479 and 2186 genes were differentially expressed (DEGs) in pollinated fruit and auxin treated fruit, respectively. In the parthenocarpic cultivar, 10,497 in pollinated fruit and 5718 in auxin treated fruit. A comparison between transcriptome of the unpollinated fruit for each cultivar has been performed determining that 6120 genes were differentially expressed. Annotation analysis of these DEGs revealed that cell cycle, regulation of transcription, carbohydrate metabolism and coordination between auxin, ethylene and gibberellin were enriched biological processes during pollinated and parthenocarpic fruit set.

**Conclusion:**

This analysis revealed the important role of hormones during fruit set, establishing the activating role of auxins and gibberellins against the inhibitory role of ethylene and different candidate genes that could be useful as markers for parthenocarpic selection in the current breeding programs of zucchini.

**Electronic supplementary material:**

The online version of this article (10.1186/s12870-019-1632-2) contains supplementary material, which is available to authorized users.

## Background

Fruit set is defined as the transition of an ovary to a growing young fruit, and depends on the successful completion of pollination and fertilization. Pollination is the transfer of a pollen grain from the anther to the stigma. Fertilization occurs in the ovule, and requires pollen tubes growth in the stylar tissue and fusion with the egg cell [1]. Both processes are affected negatively by unfavourable environmental conditions such as low/ high temperature or inadequate humidity that prevents fruit set in the majority of flowering plants [2].

In the case of zucchini, one of the most important morphotypes of *Cucurbita pepo*, these harsh conditions especially occur in off-season crops, causing economic losses due to low fruit yield. Parthenocarpy, fruit development in the absence of pollination/fertilization, has been recognized as an important trait to avoid this problem in different fruit crops [3], considering than each species shows specific responses following pollination/fertilization, In *Cucurbita*, the most practical mean of increasing fruit set of zucchini when pollination/fertilization is inadequate would be the use of cultivars with vegetative parthenocarpy, innate ability to set parthenocarpic fruit [4], but this kind of parthenocarpy is limited to a few cultivars in *C. pepo.* For several cultivars of zucchini, we provided previously an exhaustive description of the specific changes in fruit set observed after successful pollination or parthenocarpy, when compared with non-pollination [5].

Parthenocarpy has been related to certain plant hormones as auxins, gibberellins, cytokinin and brassinosteroids [6]. Exogenous application of these plant hormones induces parthenocarpy fruit set in cucumber and zucchini [4], and high levels of endogenous IAA were found in parthenocarpic fruit respect to pollinated fruit in cucumber [7]. Furthermore, natural parthenocarpic tomato mutants *pat* and *pat-2* accumulate high levels of gibberellin in the unpollinated ovaries [8].

Transcriptome analyses have led to the successful identification of some genes associated with parthenocarpy. Over-expression of *SlGA20ox1* was found at high levels throughout fruit growth in the *pat* mutant in tomato [8]. Over expression of *SlTIR1* (an auxin receptor) give rise to parthenocarpic fruit set in cucumber treated with exogenous auxins [9]. Down-regulated expression of *SlARF7* (Auxin Response Factor 7) and *SlIAA9* (*Aux/IAA* gene) also induced parthenocarpy in transgenic tomatoes [10, 11]. On the other hand, mutations in *ARF8* and *IAA9* induced parthenocarpy in *Arabidopsis* and tomato respectively [12, 13]. These genes, *ARF8*, *IAA9* and *TIR1*, showed downregulation during fruit set in pollinated fruit of non-parthenocarpic cultivars of *C. pepo.* Nevertheless, differences found in transcriptome analysis suggest that other complementary pathways could regulate fruit formation in parthenocarpic cultivars of this species [5]. Consequently, more knowledge about transcriptome responses of fruit set is required to exploit parthenocarpy in zucchini.

The reference transcriptome of *Cucurbita pepo L. ssp. pepo* is a valuable resource for identification of transcripts involved in specific biological processes, improving genome annotation, elucidating phylogenetic relationships, providing SSR and SNP markers and large-scale expression analysis [14]. Large-scale expression analysis, as microarray gene chip experiments, could be a powerful tool for monitoring the expression level of thousands of genes involved in fruit set and parthenocarpy in zucchini, but detecting transcripts by microarray technology is limited to genomic sequencing. Moreover, it is a laborious and capital intensive task, and the statistical reproducibility of the data is relatively poor. Nowadays, the development of next-generation sequencing technologies as RNA-sequencing (RNA-seq) opens a new horizon for mapping and quantifying transcriptome, creating opportunities to better understand the molecular basis of pathways that could regulate these processes. These methods improved sequencing capabilities with respect to amount of data, time, and cost, and do not depend on prior gene identification or assembly onto microarrays [15, 16]. RNA-seq technology has been used to improve the genome annotations, to detect areas of alternative splicing to discover new genes and novel transcribed regions and to perform differential expression analysis. This method has many advantages such as lower background signal, higher suitability for both known transcripts and new genes, and ability to quantify a large dynamic range of expression levels, with absolute rather than relative values, even with species that lacked a reference genome [17]. RNA-seq has been used for analyzing the transcriptome responses to fruit set and parthenocarpy in cucumber. [18]. However this type of technology has not been yet applied for studying these processes in zucchini.

In the current study, differences in fruit transcriptome of two cultivars of zucchini, a non-parthenocarpic cultivar and a parthenocarpic cultivar has been analysed through RNA-seq in an attempt to identify genes that play important roles during pollination, fruit set and parthenocarpy.

## Results

### Analysis of RNA-seq libraries

Two cultivars of Cucurbita pepo spp. pepo morphotype zucchini were used in this study, a non-parthenocarpic cultivar MUCU-16(Acronym MU16), and a parthenocarpic cultivar Whitaker (Acronym WHT). Samples of pollinated fruit (PF), non-pollinated fruit (UF) and auxin treated fruit (AF) of both cultivars were obtained to construct 6 libraries to be sequenced for RNA Seq analysis. Raw data was generated through Illumina HiSeq 2500 sequencing and was subjected to initial treatment, low quality regions and adapter sequences have been trimmed. Samples ranged from 42.4 to 84.8 million reads with mean read length of 101 pb, which is enough for the quantitative analysis of gene expression (Table 1).

**Table 1:** Statistics of the mapping of RNA-seq libraries. MU16 and WHT correspond to the genotipes MUCU-16 (Non parthenocarpic) and Whitaker (parthenocarpic) respectively. UF, PF and AF correspond to the fruit treatments and control Unpollinated Fruit, Pollinated Fruit and Auxin Treated Fruit respectively

*C. pepo* transcriptome v3	UF MU16	PF MU16	AF MU16	UF WHT	PF WHT	AF WHT
Raw Reads	80,057,152	84,843,056	42,494,528	66,404,290	64,111,386	65,846,378
Total clean reads	78,004,856 100.00%	81,811,068 100.00%	41,482,294 100.00%	64,924,704 100.00%	62,637,308 100.00%	64,790,236 100.00%
Total mapped reads	68,718,640 88.10%	71,723,387 87.67%	36,470,891 87.92%	56,826,277 87.53%	54,627,828 87.21%	56,479,396 87.17%
Unique mapped reads	40,386,479 51.77%	42,489,891 51.94%	21,235,135 51.19%	32,592,183 50.20%	32,370,915 51.68%	32,814,187 50.65%
Multiple mapped reads	28,332,161 36.32%	29,333,496 35.73%	15,235,756 36.73%	24,234,094 37.33%	22,256,913 35.53%	23,665,209 36.53%
Unmapped reads	9,286,216 11.90%	10,087,681 12.33%	5,011,403 12.08%	8,098,427 12.47%	8,009,480 12.79%	8,310,840 12.83%
New *C. pepo* transcriptome	UF MU16	PF MU16	AF MU16	UF WHT	PF WHT	AF WHT
Raw Reads	80,057,152	84,843,056	42,494,528	66,404,290	64,111,386	65,846,378
Total clean reads	78,004,856 100.00%	81,811,068 100.00%	41,482,294 100.00%	64,924,704 100.00%	62,637,308 100.00%	64,790,236 100.00%
Total mapped reads	71,334,429 91.92%	74,876,005 92.12%	37,945,731 91.92%	58,994,451 91.31%	57,271,131 91.95%	59,000,017 91.45%
Unique mapped reads	64,606,549 83.25%	67,204,342 82.68%	34,318,723 83.13%	53,882,331 83.31%	51,536,930 81.74%	53,573,732 83.04%
Multiple mapped reads	6,727,880 8.67%	7,671,663 9.44%	3,627,008 8.79%	5,172,120 8.01%	5,734,201 9.21%	5,426,285 8.41%
Unmapped reads	6,270,823 8.08%	6,404,401 7.88%	3,336,953 8.08%	5,613,463 8.69%	5,015,741 8.05%	5,517,123 8.55%

Data is represented by numbers of reads and percentage represented

The reference transcriptome of *C. pepo v3* consists of 108,062 transcripts that represent 73,239 unigenes clusters with an average length of 1052 pb. RNA-seq reads obtained from six libraries were mapped to the reference transcriptome of *C. pepo v3,* with a mean of 87% success rate of mapping, but high percentage of multiple reads (36%) were obtained (Table 1).

Therefore, CD-hit program [19] was used to analyse the redundancy of the reference transcriptome and sequences with 85% of homology were clustered. The longest unigene was chosen to represent each cluster, resulting in a total of 73,239 unigenes with an average length of 818 pb. Sequencing reads were newly mapped to the processed transcriptome, obtaining higher rate of unique mapped reads and lower number of multiple mapped reads, with a mean of 92% success rate of mapping (Table 1). This improved RNA-seq using the processed transcriptome was used for subsequent analysis.

### Differentially expressed genes during fruit set

**Fig. 1:**
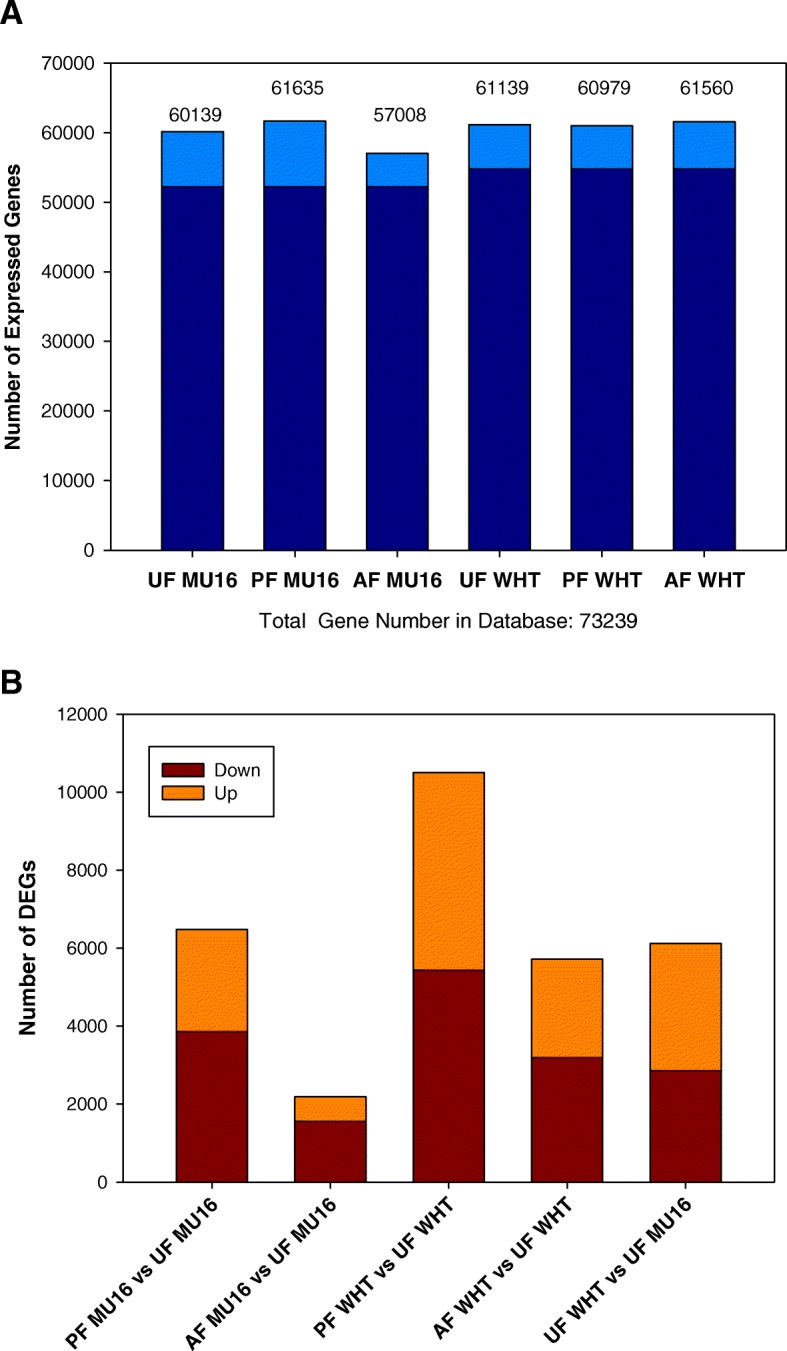
Statistics of expressed genes during fruit set in zucchini. **a** Number of detected expressed genes for each sample (light blue: different genes expressed; dark blue: common genes expressed). **b** Number of up-regulated and down-regulated DEGs (differentially expressed genes) in each pairwise comparison. MU16 and WHT correspond to the genotipes MUCU-16 (Non parthenocarpic) and Whitaker (parthenocarpic) respectively. UF, PF and AF correspond to the Fruit treatments and control Unpollinated Fruit, Pollinated Fruit and Auxin Treated Fruit respectively

RNA-seq mapping revealed the number of genes expressed in each sample (Fig. 1). Comparison between pollinated, auxin and unpollinated fruit treatments in both cultivars showed that 52,240 and 54,761 genes were expressed in common in MUCU-16 and Whitaker respectively independently of treatment applied. Genes with differential expression during fruit set were filtered using the transcriptome of unpollinated fruit for both cultivars. A FDR of 0.05 and an absolute value of fold change ≥2 were used as the threshold to judge the significance of the differential gene expression(Additional file 1) In the non-parthenocarpic cultivar, 6479 and 2186 genes were differentially expressed in pollinated fruit (PF MU16 vs UF MU16) and auxin treated fruit (AF MU16 vs UF MU16) respectively. In the parthenocarpic cultivar, 10,497 genes were differentially expressed in pollinated fruit (PF WHT vs UF WHT) and 5718 genes in auxin treated fruit (AF WHT vs UF WHT). A comparison between transcriptome of the unpollinated fruit for each cultivar has been performed (UF WHT vs UF MU16), determining that 6120 genes were differentially expressed. It was also found that pollination treatment generated the highest number of expressed genes and the highest number of differentially expressed genes (Fig. 1).

Previous *C. pepo* gene annotation was incomplete because about 50% of DEGs had previous annotation in *C. pepo* transcriptome v3. Structural and functional annotation of DEGs was performed by BLAST analysis (e-value of 1e-25) with public databases. DEGs were compared to *Arabidopsis* genome and *Arabidopsis* proteins from TAIR, Nucleotide collection nr from NCBI, Swiss-Prot database, *Cucurbita maxima genome* and *Cucurbita pepo* genome v4 (Additional file 2). About 30% of DEGs had one significant hit with *Arabidopsis* genome and more than 40% of DEGs blasted with *Arabidopsis* proteins, improving the previous annotation with this species (Table 2). Blast analysis revealed that 55% of unigenes had significant matches in Nucleotide collection nr database, where the most of hits found were with *Cucumis melo* and *Cucumis sativus* gene. Sequence homology was also performed with the accurate database Swiss-Prot, obtaining more than 30% had significant matches (Table 2). This new blast analysis allows the annotation of 20% of unigenes that did not have previously annotated in the reference *C. pepo* transcriptome. Additionally, blast analysis was carried out with the genomes of *Cucurbita maxima* and *Cucurbita pepo* (Additional file 2).

**Table 2:** Functional annotation statistics

	PF MU16 vs UF MU16	PF MU16 vs UF MU16	PF MU16 vs UF MU16	PF MU16 vs UF MU16	PF MU16 vs UF MU16
Database					
*C. pepo* trancriptome v3	3539 54.62%	1249 57.14%	6231 59.36%	3354 58.66%	3261 53.28%
*A. thaliana* genome	2177 33.60%	734 33.58%	3951 37.64%	1988 34.77%	1826 29.84%
*A. thaliana* péptides	2875 44.37%	995 45.52%	5122 48.79%	2701 47.24%	2564 41.90%
NCBI Nucleotide collection nr	3581 55.27%	1255 57.41%	6276 59.79%	3748 65.55%	3194 52.19%
Swiss prot	2398 37.01%	850 38.88%	4226 40.26%	2238 39.14%	2142 35.00%
*Cucurbita pepo ssp.ovifera*	3489 53.85%	1273 58.23%	5878 56.00	3247 56.79%	3331 54.43%
IntrePro	2659 41.04%	1367 62.53%	6805 64.83%	3671 64.20%	3793 61.98%
Gene Ontology GO term	1650 25.47%	623 28.50%	3089 29.43%	1632 28.54%	1598 26.11%
KEGG Pathway	275 4.24%	114 5.22%	564 5.37%	312 5.46%	286 4.67%

Number and percentage of DEGs annotated in each database

Gene Ontology (GO) terms were further assigned to screened DEGs (Additional file 3) based on their sequence similarities to domains they contain in Interpro database. Over 25% of DEGs were assigned at least one GO term in biological process, molecular function and cellular component categories (Table 3). These unigenes, were further classified into different functional categories using a set of plant-specific GO slims (Additional file 3). Functional classifications of DEGs into plant specific GO slims (level 2) within the biological process, molecular function and cellular component categories were carried out. Metabolic process and cellular process were the most highly represented groups in biological process (Table 3). Genes involved in other important biological processes such as response to stimulus were also identified in pollination in the non-parthenocarpic cultivar and single-organism process in parthenocarpic cultivar. Under molecular function category, assignments were to the binding and catalytic activities (Table 3). It is worth noting that GO annotations revealed high number of transferases, kinases and hydrolases in this category, suggesting that genes involved in the secondary metabolite synthesis pathways were induced during fruit set (Additional file 3). In the case of cellular component, GO terms related to cell, membrane and organelle were well-represented (Table 3) DEGs also were annotated using KEGG pathway database (Additional file 3).

**Table 3:** Classification of DEGs during fruit set into Gene Ontology (GO)

	PF MU16 vs	AF MU16 vs	PF WHT vs	AF WHT vs	UF WHT vs
	UF MU16	UF MU16	UF WHT	UF WHT	UFMU16
GO Biological Process (%)					
metabolic process	52.88	58.47	51.90	52.90	53.55
cellular process	40.22	41.53	41.25	39.99	38.85
response to stimulus	6.91	–	–	–	–
single-organism process	–	–	6.85	7.12	7.61
GO Molecular function (%)					
binding	57.43	56.46	56.59	54.65	55.15
catalytic activity	42.57	43.54	43.41	45.35	44.85
GO Cellular Component (%)					
cell	27.58	23.39	28.79	26.33	26.53
cell part	27.58	23.39	28.72	26.33	26.53
membrane	25.62	38.53	23.17	30.59	30.68
organelle	19.22	14.68	19.32	16.74	16.25

**Fig. 2:**
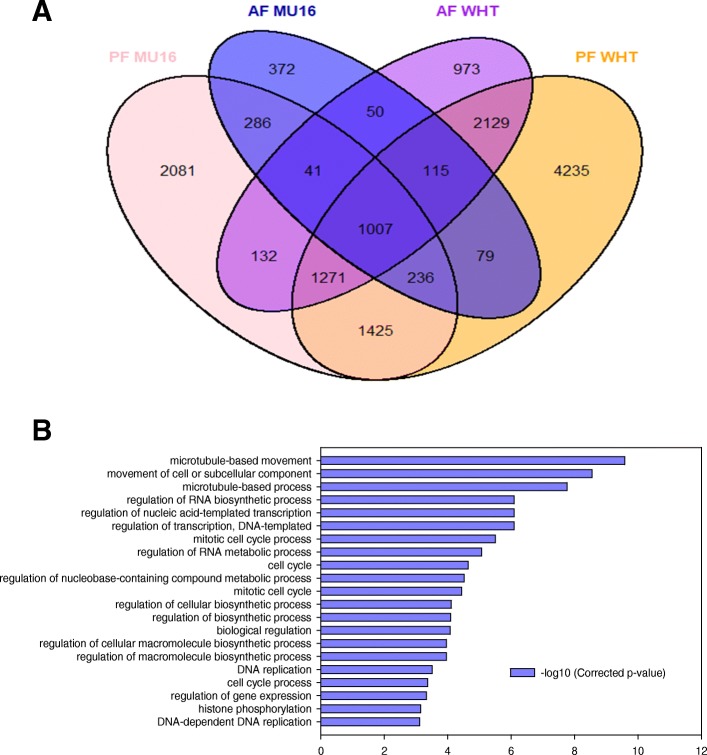
**a** Venn diagram of DEGs associated with fruit set. Common and distinct DEGs in pollinated fruit (PF) and auxin treated fruit (AF) with respect to unpollinated fruit in each cultivar, MUCU-16 (MU16) and Whitaker (WHT) (**b**) GO enrichment in the category of biological process of common DEGs during fruit set of *C. pepo*

DEGs between pollination and auxin treatment in both cultivars were compared. Cluster analysis was used to screen the common expressed genes between them. A total of 1570 and 4522 were induced in both treatments in MUCU-16 and Whitaker, respectively (Fig. 2a). Of these subsets of DEGs, 1007 genes were common in both cultivars, which represented 64% of common DEGs in MUCU-16 cultivar and 22% in Whitaker cultivar. To further understand the function of these DEGs, GO enrichment analysis in the category of biological process was performed (*p* < 0.05). DEGs involved in cell cycle, DNA replications, microtubule movement, regulation of transcription or biosynthetic process were highly enriched (Fig. 2b).

**Fig. 3:**
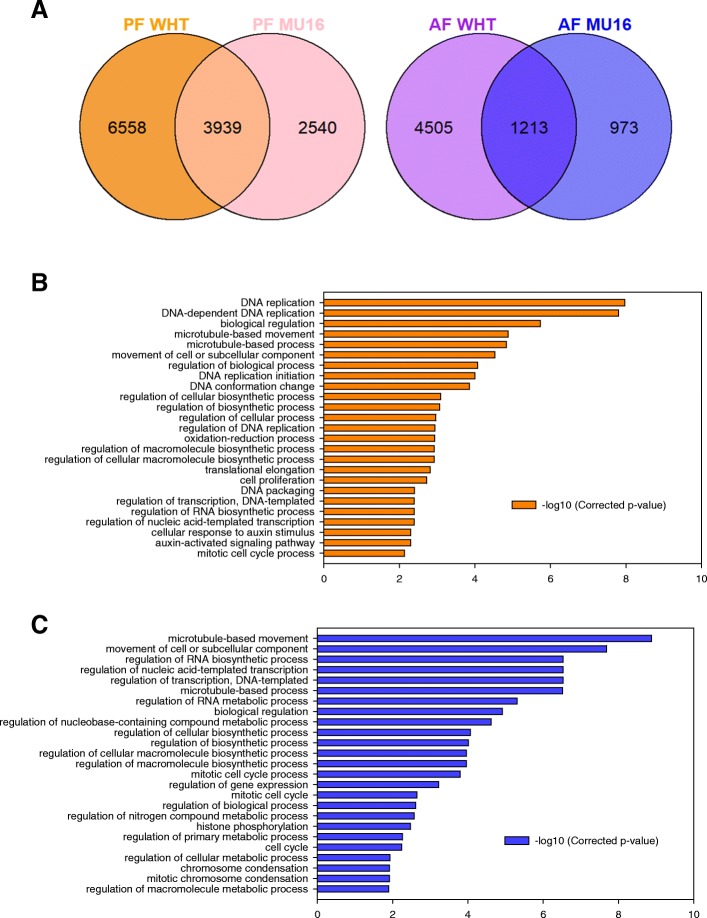
**(a)** Venn diagram of DEGs associated with fruit set dependent of pollination (orange and pink) and auxin treatment (purple and blue). Common and distinct DEGs in pollinated fruit (PF) and auxin treat fruit (AF) respect to unpollinated fruit in each cultivar. **(b)** GO enrichment in the category of biological process of 3939 DEGs expressed in common during fruit set dependent of pollination in cultivars, MUCU-16 (MU16) and Whitaker (WHT) **(c)** GO enrichment in the category of biological process of 1213 DEGs expressed in common during fruit set dependent of auxin treatment in both cultivars

It was also compared pollination treatment between both cultivars. 3939 DEGs were induced during pollination fruit set in both cultivars (Fig. 3a). GO enrichment analysis revealed that DNA replication, microtubule movement, regulation of biosynthetic process, regulation of biological process, cell proliferation, auxin-activated signalling pathway and cellular response to auxin stimulus were enriched processes in the category of biological process (Fig. 3b). On the other hand, 1213 DEGs were induced during auxin treated fruit set in both cultivars (Fig. 3a). GO enrichment analysis showed differences in biological processes activated during auxin treatment in comparison to pollination (Fig. 3). Microtubule movement, regulation of biosynthetic process and regulation of biological process were also induced, but processes related to auxins were not enriched in this treatment (Fig. 3c).

### Expression of genes associated with cell division during fruit set

Cell division related genes were analysed based on previous DEGs results, cyclins and expansins (*EXPs*) were filtered. During pollination, 18 and 32 cyclins were induced in MUCU-16 and Whitaker, respectively. Of those cyclins, 16 DEGs were expressed in common in both cultivars, showing the same regulation. It was found 6 cyclins of type A, 4 cyclins of type B, 2 cyclins of type D, 2 cyclins of type P, *CSK1* and *CDKB2* genes. All cyclins were up-regulated during pollination, except *CYCP2–1.*Pollination also induced differential expression of expansins, and the majority of these genes were up-regulated. 4 expansins were expressed in common during pollinated fruit set in both cultivars, *EXPA1*, *EXPA8*, *EXPA4* were upregulated and *EXPLB1* was strongly downregulated (Additional file 4). During auxin treatment, 2 cyclins were found in the non-parthenocarpic cultivar. These cyclins were also induced in pollination treatment in this cultivar. In the case of the parthenocarpic cultivar, 24 cyclins were differentially expressed, activating also cyclins of type A, B, D and P. All of these cyclins were also induced during pollination in this cultivar, but its fold change was lower in auxin treatment. In the case of expansins, *EXPLB1* was also strongly downregulated in both cultivars and higher number of expansins were induced in Whitaker in comparison to MUCU-16 (Additional file 4). On the other hand, Cyclin D6–1 and *EXPLA1s*howed the same regulation and pollination/auxin treatment fruit set and during parthenocarpic fruit set (UF WHT vs UF MU16) (Additional file 4).

### Identification of transcription factors related to fruit set

**Fig. 4:**
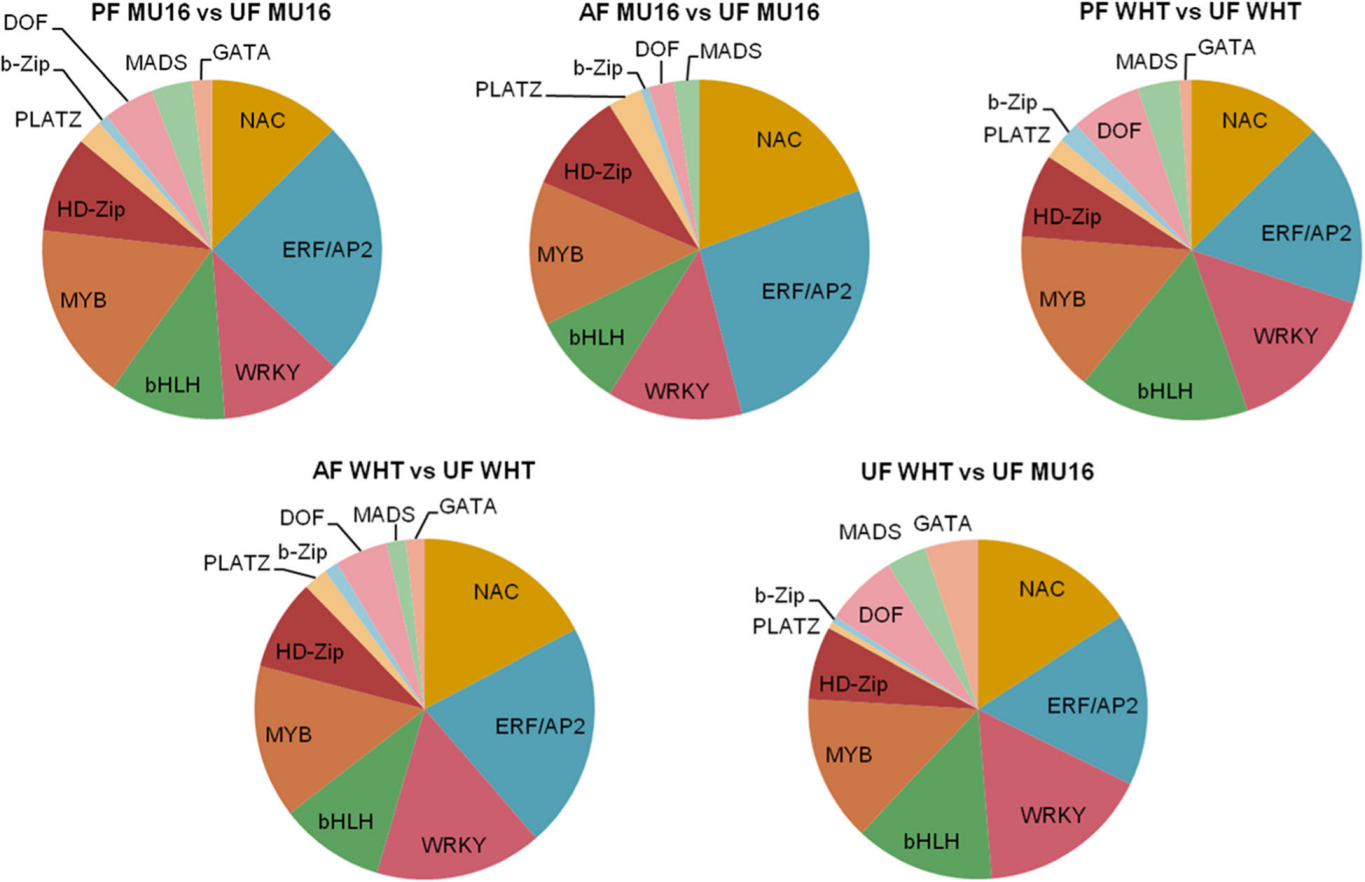
TFs families involved in zucchini fruit set. NAC (NAC domain-containing protein), ERF/AP2 (ethylene responsive factor), WRKY (WRKY DNA-binding protein), bHLH (Basic helix-loop-helix protein), MYB (Myb-related protein), HD-zip (Homeobox-leucine zipper protein), PLATZ (plant AT-rich sequence- and zinc-binding protein) b-Zip (Basic leucine zipper), DOF(DNA-binding One Zinc Finger protein), MADS (MADS-box protein) and GATA transcription factors

RNA-seq data showed that 207 and 254 transcription factors (TFs) displayed differential expression during pollination in MUCU-16 and Whitaker, respectively. In the case of auxin treatment, 124 and 220 were induced, a lower number in comparison to pollination treatment (Additional file 5). TFs were classified into several families including NAC, ERFs (Ethylene-responsive transcription factor)**,** WRKY, bHLH (Basic helix-loop-helix protein), MYB, HD-Zip (Homeobox-leucine zipper protein) and MADS-box, among which NAC, AP2/ERF and WRKY recruited the major members. The vast majority of them were downregulated after pollination and auxin treatment (Fig. 4, Additional file 5). In the case of UF WHT vs UF MU16, 158 TFs were differentially expressed. Expression of these genes was different in comparison to pollination and auxin treatment (Additional file 5).

MADS-box and HD-Zip families were reviewed in detail. MADS-box family proteins, including Agamous-like MADS box proteins (*AGL*), represented lower rate in comparison to NAC, AP2/ERF and WRKY families during fruit set (Fig. 4). DEGs analysis revealed differential expression of 6 MADS-box genes during pollinated fruit set, *CMB1* (also called *AGL3*), *SOC1* (also called *AGL20*), *AGL8*, *AGL11*, *AGL16* and *AGL18* in the parthenocarpic variety. *CMB1*, *SOC1*, *SVP*, *FBP24*, *AGL12* and *AGL16* were expressed in the non-parthenocarpic variety. *CMB1*, *SOC1* and *AGL16* were commonly downregulated during pollinated fruit set in both cultivars. During fruit set dependent of auxin treatment, *AGL8*, *AGL16* and *FBP24* were strongly downregulated in MUCU-16 cultivar. Downregulation was also observed in *AGL19* and *SOC1* in Whitaker cultivar (Additional file 5).

On the other hand, HD-zip family represented greater rate than MADS-box family (Fig. 4). The overwhelming majority of HD-zip genes were down-regulated in pollination and auxin treatment. *HAT5*, *HOX11*, *HOX20*, *ATHB7*, *ATHB12, ATBH21* and *ATHB40* were expressed in common in both cultivars during pollination and auxin treatment. Stronger downregulation was observed in *ATHB7* and *ATBH21*, more than 20 fold of expression (Additional file 5).

### Comparison of the carbohydrate-related responses during fruit set

**Fig. 5:**
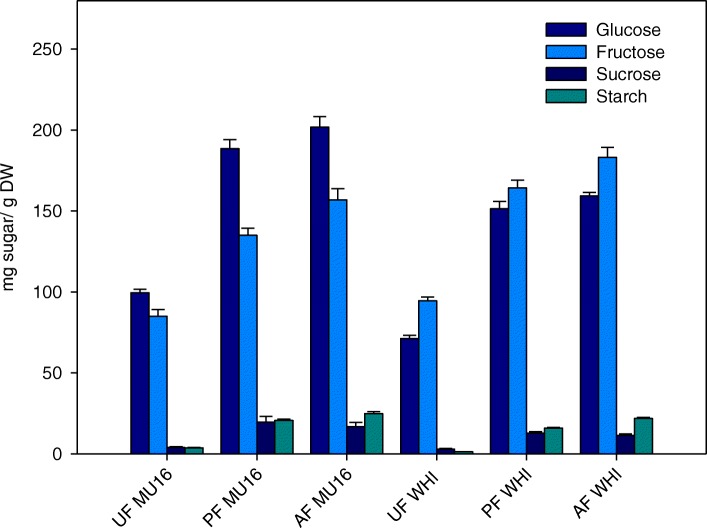
Carbohydrate content in zucchini fruits expressed in mg g^− 1^ dry weight

Carbohydrate metabolism was strongly induced during fruit set in zucchini (Additional file 6). During pollinated fruit set, 45 genes were expressed in common in both cultivars, 17 genes were up-regulated and 28 genes were down-regulated. Over-expression was found in key enzymes involved in glycolisis as enolase1, glyceraldehyde-3-phosphate dehydrogenase and fructose-bisphosphate aldolase, and genes related to starch accumulation as phosphoglucan phosphatase amyloplastic, and starch biosynthesis as 1,4-alpha-glucan-branching enzyme. On the other hand, negative regulation was found in gluconokinase, phosphoenolpyruvate carboxykinase, sucrose synthase, amylases and alpha-trehalose-phosphate synthases (Additional file 6). High levels of glucose and fructose found during pollinated fruit set in both cultivars and low levels of sucrose are produced by activation of glycolisis via glyceraldehyde-3-phosphate dehydrogenase and fructose-bisphosphate aldolase and downregulation of sucrose synthase (Fig. 5). In addition, it was also found low levels of starch in spite of activation of enzymes related to starch biosinthesis (Fig. 5, Additional file 6). During auxin treatment, 26 DEGs were expressed in common in both cultivars. Of this subset of genes, 1,4-alpha-glucan-branching enzyme and polygalacturase were up-regulated in common in both cultivars in contrast to amylases and threalose phosphate synthases, which were down-regulated. A detailed analysis of auxin treatment in each cultivar was carried out. In MUCU-16, fructose 6-phosphate 1-phosphotransferase was up-regulated. On the other hand, pyruvate kinase and phosphoenolpyruvate carboxykinase 1 were up-regulated in Whitaker (Additional file 6). Over-expression of these enzymes corroborated high levels of glucose and fructose found during auxin treated fruit set in both cultivars (Fig. 5). Nevertheless, during parthenocarpic fruit set, glucose and fructose levels were similar to unpollinated fruit of MUCU-16 (Fig. 5). This is contrary to over-expression of fructose-1,6-bisphosphatase, pyruvate kinase, glyceraldehyde-3-phosphate dehydrogenase, sucrose synthase and 6-phosphofructokinase 5, that indicated the carbohydrate biosynthesis (Additional file 6).

### Hormone metabolism and signalling during fruit set

**Fig. 6:**
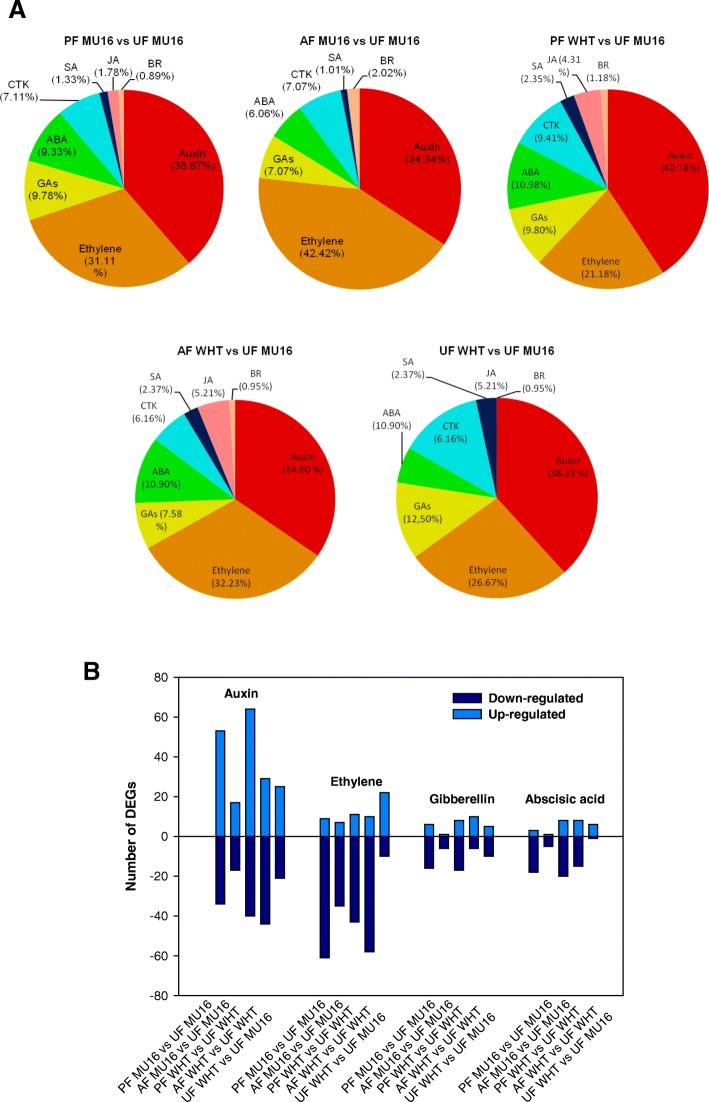
Hormone signalling during zucchini fruit set. **a** Pie charts show percentages of DEGs related to hormones: auxins, ethylene, gibberellins (GAs), abscisic acid (ABA), cytokinin (CTK), brassinosteroids (BRs), jasmonic acid (JAs) and salicylic acid (SA). **b** Columns indicate the numbers of up- and down-regulated DEGs of the most representative hormones related to fruit set

Hormone related genes were filtered based on previous DEGs results, 225 and 255 DEGs related to hormone were screened in pollinated fruit in MUCU-16 and Whitaker, respectively. However, lower number of DEGs, 99 and 210, was expressed during auxin treatment. These screened genes were involved in biosynthesis and signalling of auxins, ethylene, gibberellins, abscisic acid, cytokinin, brassinosteroids, jasmonic and salicylic acid (Additional file 7). Percentages of DEGs related to these eight hormones, number and category, were similar between treatments except in the case of auxin treatment of MUCU-16 (Fig. 6). During fruit set and parthenocarpic fruit set, the most representative hormone-related DEGs were auxins, followed by ethylene, gibberellins and abscisic acid. Regulation of gene expression was also analysed showing that DEGs related to auxins displayed higher number of up-regulated genes in comparison with ethylene, gibberellins and abscisic acid (Fig. 6).

**Fig. 7:**
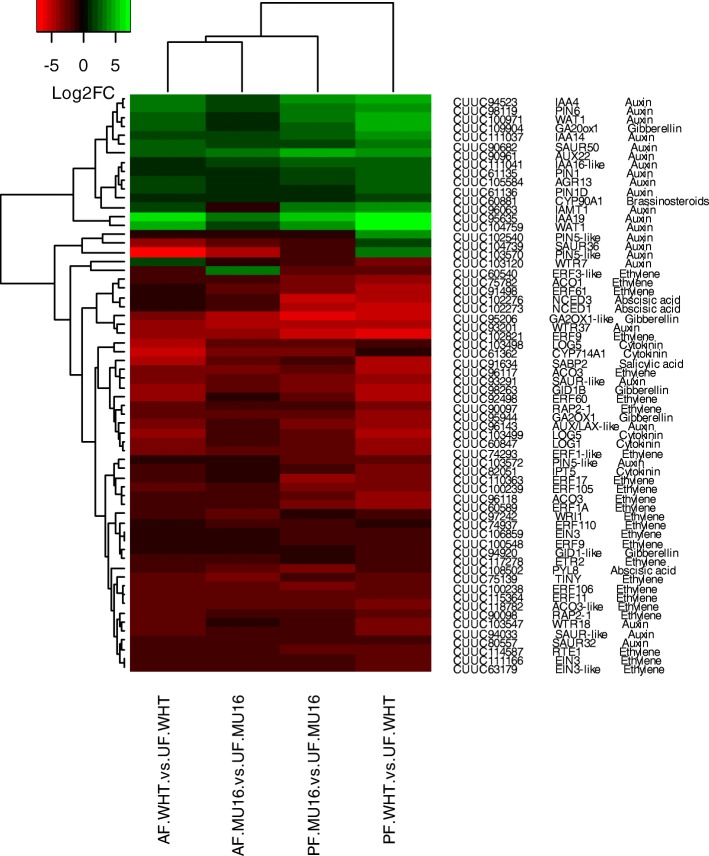
Cluster analysis of common DEGs associated with hormone biosynthesis and signalling during fruit set. Genes have been grouped based on Euclidean algorithm of Log2 Fold Change Value of differential expression analysis. Genes have been indicated as accession in *C. pepo* transcriptome, gene annotation (blast analysis) and related hormone

Cluster analysis was carried out to elucidate the regulation of common hormone-related genes during fruit set. Of the subsets of DEGs related to hormone analysed previously, 64 genes were common during pollination and auxin treatment in both cultivars. Regulation of gene expression was similar during pollination in both cultivars and during auxin treatment in both cultivars (Fig. 7). Most of these common genes were related to auxin (24 genes, 37.5%) and ethylene (25 genes, 39%). Auxin genes were involved in auxin signalling (Aux/IAA and WTR families), auxin polar transport (*PIN* Auxin efflux carriers), response to auxin (*SAUR* Auxin responsive proteins) and auxin homeostasis (indole-3-acetic acid-amido synthetase, GH3.1, and indole-3-acetate o-methyltransferase 1, IAMT1). Expression of *IAA* genes, *IAA4*, *IAA14* and *IAA16* clustered together with *PIN6*, *WAT1* and *SAUR50,* showing over-expression. However, *GH3.1,* an auxin biosynthesis gene, and others members of *SAUR family (SAUR32)* and *PIN* family (*PIN5*) decreased their expression under pollination and auxin treatment. Remarkably, *IAA19*, *AUX22* and *WAT1* showed the strongest up-regulation during fruit set. On the other hand, DEG analysis showed a decrease in mRNA levels of ethylene related genes. Ethylene biosynthesis genes (ACC oxidases, *ACO1* and *ACO3*) and ethylene signalling genes, including *ERFs* (12 *ERFs*), *EIN3* (Ethylene insensitive 3) or *ETR2* (Ethylene receptor 2) among others, displayed down-regulation during fruit set. Apart from these findings related to auxin and ethylene, a subset of genes associated with GAs biosynthesis and signalling has been also modulated during fruit set. Expression of *GA20ox1* (Gibberellin 20 oxidase 1) was found to be strongly up-regulated in contrast to expression of *GA2ox2* (Gibberellin 2-beta-dioxygenase 2) and *GID1B* (Gibberellin receptor). To a lesser extent, genes related to cytokinin (Cytokinin riboside 5′-monophosphate phosphoribohydrolases, LOG1 and LOG5, and Cytokinin synthase IPT5)*,* abscisic acid (9-cis-epoxycarotenoid dioxygenases, NCED1 and NCDE3, and abscisic acid receptor *PYL8*), brassinosteroids (Cytochrome P450 90A1, *CYP90A1*) and salicylic acid (Salicylic acid-binding protein 2, *SABP2*) were differentially expressed in common. All genes were downregulated, except *CYP90A1*. *CYP90A1*, a BRs biosynthesis gene, was up-regulated and clustered together auxin genes (Fig. 7).

### Auxin at the transcriptome level during zucchini fruit set

Genes related to auxin have been filtered during pollination and during auxin treatment in both cultivars, indicating that DEGs related to auxin biosynthesis, auxin metabolism and signalling were strongly induced during fruit set (Fig. 6, Fig. 7 and Additional file 7).

During pollinated fruit set, auxin biosynthesis genes were altered. *YUCCA6* (indole-3-pyruvate monooxygenase) and *IAMT1* were up-regulated and three genes of GH3 family, *GH3.1*, *GH3.5* and *GH3.6*, were downregulated. Related to auxin signalling, ten Aux/IAAs were expressed differently, being only *IAA26* and *IAA18* down-regulated in both cultivars. Contrasting with Aux/IAA family, ARFs showed different regulation between cultivars, ARF5 and ARF16 were down-regulated in MUCU-16 cultivar, and *ARF5*, *ARF7* and *ARF18* were up-regulated in Whitaker cultivar. Over-expression was also found in auxin efflux carriers, *PIN1A*, *PIN1B*, *PIN6*, *PIN8*, and auxin influx carriers, *AUX1*, *LAX2* and *LAX4* (Additional file 7).

During auxin treatment, auxin biosynthesis genes were also differentially expressed, *YUCCA8* was downregulated in MUCU-16, and *YUCCA7* and *IAMT1* was up-regulated in Whitaker. IAA and ARF genes were also modulated during auxin treatment and, most of them showed the same regulation than during pollinated fruit set. IAA4, IAA14, IAA16 and IAA19 were also up-regulated during auxin treated fruit set in both cultivars, but *ARF5* and *ARF18* were only up-regulated in Whitaker cultivar. Auxin treatment also produced changes in PIN proteins and AUX/LAX proteins. *PIN1*, *PIN1D* and *PIN6* were over-expressed together two AUX/LAX transporters, indicating that efflux and influx of auxins also occurred in auxin treatment. In the case of parthenocarpic fruit set (UF WHT vs UF MU16 comparison), it was found DEGs related to auxin with similar expression to pollination or auxin treatment. *ARF18*, *PIN1B*, *PIN8* and *LAX1* were also upregulated as pollination/auxin treatment (Additional file 7).

### Biosynthesis and signalling of ethylene and gibberellin during fruit set

Ethylene metabolism is one of the most represented hormones during pollinated fruit set. RNA-Seq data revealed a decrease in the mRNA levels of most of ethylene related gene (Fig. 6, Fig. 7, Additional file 7). *ACO1* and *ACO3*, which catalyze the subsequent steps of ethylene biosynthesis via S-adenosyl-L-methionine, were strongly downregulated during pollinated fruit set in contrast to auxin biosynthesis genes. As occurred for ACO genes, ethylene signalling genes including *ERFs*, *ETR2* and *EIN3s* appeared down-regulated during pollinated fruit set. Higher number of DEGs related to ethylene was also altered during auxin treatment, and their regulation was similar to found during pollinated fruit set (Fig. 7, Additional file 7).

During parthenocarpic fruit set, *ACO1*, *ETR2* and five *ERFs* (*ERF2, ERF11, ERF12,* ERF17 and *ERF60*) were downregulated as pollination/ auxin treatment in both cultivars. Moreover, *WIN1* were upregulated as pollination treatment in Whitaker cultivar (Additional file 7).

A subset of genes associated with GAs biosynthesis and signalling has also been modulated during pollinated fruit set (Table 4). Three GID1-like genes, that encode GA receptors, showed down-regulation in response to pollination as well as *GID2*, an F-box protein that is essential for GA-mediated DELLA protein degradation. *GA2ox1* and *GA2ox8*, which play important role in gibberellins catabolism, displayed a decrease of gene expression. However, *GA3OX4*, involved in production of GA bioactive for reproductive development, and *GASA4*, gibberellin-regulated protein, was up-regulated during pollination. Remarkably, genes related to gibberellin biosynthesis, *KAO1* (Ent-kaurenoic acid oxidase 1) and *KAO2* (Ent-kaurenoic acid oxidase 2), which catalyze three successive oxidations of ent-kaurenoic acid giving gibberellin 12 (GA12), and *GA20ox1* and *GA20ox2*, key enzymes of gibberellin biosynthesis that catalyzes the conversion of GA12 and GA53 to GA9 and GA20 respectively, were up-regulated during pollinated fruit set in zucchini. Five of these genes were also common between cultivars during auxin treatment (Table 4). In the case of parthenocarpic fruit set, 15 genes related to gibberellin were differentially expressed (Additional file 7). Of this subset of genes, four genes showed the same regulation as pollinated/ auxin treated fruit set. *GA20ox1* and *GASA4* were up-regulated in contrast to *GA2ox8* and *GID1B* (Table 4).

**Table 4:** Expression of common DEGs related to gibberellins signalling and biosynthesis during fruit set in zucchini

	PF MU16 vs UF MU16	AF MU16 vs UF MU16	PF WHT vs UF WHT	AF WHT vs UF WHT	UF WHT vs UF MU16
GA20ox2-like	−1.33		−1.87	− 1.19	
GID1C	−1.37		−1.22		
GA2ox8	−2.73	−1.53	−2.66		−3.41
GA20ox1	2.72	1.39	4.61	2.49	3.50
KO1	−1.46		−1.38		
GA20ox2	2.06		3.46	1.56	−1.16
GA3ox4	1.49		2.19	1.20	
F-box GID2	−1.20		− 1.14		
GASA4	1.39		2.95	2.06	3.11
GID1B-like	−1.38	−1.38	−1.88	−2.65	
GA2ox1-like	−5.81	−4.95	−5.77	−2.65	
GA2ox1	−2.26	−2.45	−3.88	1.85	1.95
KAO2	1.97		2.48	−1.02	
KAO1	2.04		2.53		
GA2ox8-like	−1.63		− 1.73	3.12	
GID1B	−3.12	−2.85	−4.85	1.04	−1.18

Genes Expression is represented by Log_2_ Fold Change

## Discussion

### A modification in *C. pepo* transcriptome allows more specific mapping during gene expression analysis in RNA-seq

RNA-seq is a high-throughput sequencing assay that combines transcript identification and quantification of gene expression, core activities to study many processes in the plant life cycle as fruit set. Two strategies are possible to read mapping and transcript identification when a reference sequence is available, genome mapping or transcriptome mapping [17]. In the case of this assay, mapping was carried out against a reference transcriptome since the new version of *C. pepo* genome (https://bioinf.comav.upv.es/downloads/zucchini/) lacked of gene annotation [20].

In order to reduce the high percentage of multiple mapped reads and unmapped reads that could limit gene identification (Table 1), transcriptome sequenced from root, leaf and flower tissue was modified. This processed transcriptome improved mapping statistics of RNA-seq libraries. Total mapped reads were higher (Table 1) and similar to RNA-seq analysis using the last version of genome [20].

Previous gene annotation of the reference *C. pepo* transcriptome was also improved by a new blast analysis of DEGs with a more restrictive e-value (cut off e-value of 1e-25) carried out in this work, increasing the numbers of genes with functional annotation in the processed *C. pepo* transcriptome (Table 2, Additional file 2). In spite of this deeply blast analysis, there are still a number of non-annotated transcripts in *C. pepo* transcriptome (Additional file 2). These transcripts without annotation may be consequence to the short sequences generated by the 454 sequencing technology used to assembly the first transcriptome of *C. pepo* [14]. Probably, these sequences lack the conserved functional domains or may be no-codings RNAs (Additional file 2).

### Cluster analysis of DEGs elucidated differences between pollinated fruit set and auxin treated fruit set

Comparative analysis of gene expressed showed a high number of common expressed genes between pollinated, auxin treated and unpollinated fruit in zucchini, indicating that there are common pathways between treatments (Fig. 1). Thus, it was necessary to carry out DEG analysis to find key role genes induced by pollination and/or auxin treatment during fruit set [17]. DEGs analysis revealed that pollination induced the highest number of DEGs and the highest values of fold change (Fig. 1, Additional file 1), although 1007 DEGs were expressed in common during pollination and auxin treatment (Fig. 2), which revealed that rapid activation of fruit metabolism is mediated by cell cycle, regulation of transcription, regulation of biosynthetic process or DNA replication (Fig. 2). However, DEGs cluster analysis and GO enrichment analysis revealed differences between pollinated fruit set and auxin treated fruit set. Pollination activated processes related to auxins as auxin-activated signalling pathway and cellular response to auxin stimulus, not found during auxin treated fruit set. On the other hand, auxin treatment activated many more processes related to cell division, not enriched during pollinated fruit set (Fig. 3). This corroborates the hypothesis that the addition of synthetic hormones does not initiate the same processes as natural pollination [11, 21].

### Cell division is an enriched biological process during fruit set

During early fruit development in zucchini, fruit length and placental diameter grow rapidly suggesting that cell division is increased [5]. Moreover, it was found that functional categories of DEGs by GO term enrichment analysis showed that cell cycle, cell cycle progress, mitotic cell cycle and mitotic cell cycle progress related genes were strongly induced (Fig. 2). Differential expression observed in cyclins, CDKs (cyclin dependent kinase enzymes) and cell wall genes during zucchini fruit set positively correlated with rapid cell division observed in zucchini, and also observed during early fruit development of tomato and cucumber [22, 23].

Cyclins are the most important cell cycle regulators and control the progression of cells through the cell cycle by activating CDKs [24]. D-type cyclins (*CYCDs*) that regulate the G1–S transition and G2-M transition [25]. A-type cyclins (*CYCAs*), that are mainly present from S phase to M phase, and B-type cyclins (*CYCBs*), that take part the G2–M transition and during M phase [26], were differentially expressed, evidencing a cell cycle progression during fruit set which induce the exponential growth phase observed previously [5]. Of these subfamilies of cyclins, cyclin D proteins are often noted as sensors of environment, linking hormonal signal with external conditions, and reporting to cell on the favourable conditions for cell-cycle start [27]. D-type cyclins as *CYCD3;1*, *CYCD3;2* and *CYCD5;1* were over-expressed during zucchini fruit set, (Additional file 4), and similar regulation had already been previously described in cucumber fruit [23] which would indicate their fundamental role in fruit set process. It is particularly important to point out that *CYCD3;1* was reported to be a rate-limiting factor for G1 /S transition [25] and its expression is regulated by hormones signalling and the availability of nutrients as sugars Previous results reported that *CYCD3;1* activated cell division at the G1-S cell cycle phase transition in response to cytokinin and auxin in *Arabidopsis* [28]. Over-expression of this gene found in zucchini fruit could be related to over-expression of auxin and cytokinin biosynthesis and signalling genes also found (Fig. 7). Moreover, *CYCD6;1*, a cyclin involved G1-S cell cycle phase transition, were over-expressed during pollinated/auxin treatment and parthenocarpic fruit set (Additional file 4), indicating that its over expression is closely linked to parthenocarpy in zucchini. Together cyclins, *CDKs* regulate cell cycle through the association of these proteins to two key classes of regulatory molecules, *CKS1* and *CKS2* (Cyclin-dependent kinases regulatory subunits) [25]. In the case of zucchini, *CKS1* and *CDKB2;2* were over-expressed with similar fold change during fruit set (Additional file 4),. These proteins have shown interaction between them in *Arabidopsis,* and this interaction may also occur in zucchini, regulating cell cycle [29].

Cell cycle progression gives rise to the formation of two daughter cells. Prior to this process, cell undergoes a rapid enlargement (cell expansion) that involves the wall loosening. Expansins have been recognized as the major wall-loosening agents and cause extension of plant cell walls by disrupting non-covalent binding between cellulose and hemicelluloses [30]. DEG analysis revealed expasins of the four subfamilies identified in plants, A-expansins (*EXPAs*), B-expansins (*EXPBs*), expansin-like A (*EXLA*) and expansin-like B (*EXLB*) during zucchini fruit set (Additional file 4). Previous studies showed that expansins were also highly enriched in growing cucumber fruits with similar differential expression to found in zucchini fruits, *EXPA1* and *EXPA8* were up-regulated in contrast to the strongly downregulation showed by *EXPLB1* [23]. Interestingly, expansins are thought to linked cell enlargement and cell wall changes with auxin signalling [25]. Over-expression of *EXPA1* and *EXPA8* found in zucchini fruit might be induced by auxin signalling in *Arabidopsis* [31]. The relation between expression of expansins and auxin signalling in zucchini could be underline by downregulation of *ARF7* during pollinated fruit set (Additional file 7). Arabidopsis mutants in ARF7 (loss of function mutant) showed less expression level of *EXPA1* and *EXPA8* [31].

### Down-regulation of MADS-box and HD-ZIP transcription factors play key roles in zucchini fruit set

Gene expression at the level of transcription is mediated by transcription factors and is crucial for almost all biological processes, including fruit development. Different TFs were differentially expressed during fruit set, being NAC, ERF/AP2, WRKY and bhLH the most representative families (Fig. 4), similar to found during siliques development in *Arabidopsis* and fruit tomato [22, 32]. This indicates that rate between the different families of TFs could affect fruit development in zucchini.

A further focus was made on the MADS-box and HD-ZIP families, which play key roles in fruit, set, via down-regulating their transcription in tomato and *Arabidopsis*, and contain many members that are functionally well-characterized during fruit development [22, 32]. TF genes in this group are AGAMOUS like genes, MADS-box genes whose expression diminished in developing ovules when the integuments appear in *Arabidopsis* [32]. In the case of zucchini, when the fruit is pollinated in both cultivars, three AGAMOUS like genes were downregulated, *AGL16*, *SOC1* (*AGL20*), *CMB1* (*AGL3*), indicating the development of fertilized ovules. Particularly, downregulation of *AGL16*, involved in repression of floral transition, suggested that the transition from floral stage to fruit stage occurred [32]. However, there were no common genes between cultivars during auxin treatment suggesting that different pathways related to MADS-box proteins were induced (Additional file 5).

TFs of HD-Zip family reduced their expression during zucchini fruit set as previously reported in tomato fruit [22]. Downregulation of *HAT5* and strong downregulation of *ATHB-40* were also occurred in tomato fruit (Additional file 5), showing that these genes could play key roles in fruit development regulation [22]. In addition, this downregulation may be mediated by hormones accumulation in fruit tissues induced by pollination or auxin treatment [7]. Increased levels of auxins and ABA during fruit set may produce downregulation of *ATHB-40* [33] and *ATHB7* [34], respectively.

### Carbohydrate metabolism is induced by pollination and auxin treatment

Carbohydrates are major category of compounds that include among others: reducing (glucose and fructose), non reducing (sucrose) sugars, starches and cellulose which play an important role in the structure, function of all cells and are crucial factors that influence fruit quality [35, 36]. High levels of glucose and fructose were found after pollination and auxin treatment in zucchini fruit, indicating that the imported photoassimilates becoming mainly glucose and fructose in these first phases of fruit development and to a lesser extend to sucrose and starch (Fig. 5). Increased of these carbohydrates could be a consequence of glycolisis activation by enolase 1, glyceraldehyde-3-phosphate dehydrogenase and fructose-bisphosphate aldolase during pollinated fruit set [18]. Enolase 1 synthesizes pyruvate from D-glyceraldehyde 3-phosphate [37], glyceraldehyde-3-phosphate dehydrogenase (GAPC-1) catalyzes the first step of the pathway by converting D-glyceraldehyde 3-phosphate into 3-phospho-D-glyceroyl phosphate [38], and fructose-bisphosphate aldolase is involved in a subpathway that synthesizes D-glyceraldehyde 3-phosphate and glycerone phosphate from D-glucose (Additional file 6) [39]. These enzymes are essential not only for carbohydrate metabolism but also for proper fruit development, because they can regulate seed and fruit development. *Gapc-1* null mutant showed low seed number, altered embryo development and apical morphological alterations in siliques in Arabidopsis [38]. Thus, its over-expression may be caused the proper fruit growth observed in pollinated fruit in zucchini [5].

In the case of auxin treatment, high levels of fructose and glucose may be due to the activation of fructose 6-phosphate 1-phosphotransferase, pyruvate kinase and phosphoenolpyruvate carboxykinase 1, enzymes also involved in glycolisis and gluconeogenesis [35]. Fructose 6-phosphate 1-phosphotransferase catalyzes the phosphorylation of D-fructose 6-phosphate and can thus function both in glycolysis and gluconeogenesis [40], and pyruvate kinase catalyses the irreversible synthesis of pyruvate and ATP, which are both used in multiple biochemical pathways [41].

Complex carbohydrates as starch are formed by linkage of monosaccharides as glucose and fructose [36], however low levels of starch were found during pollinated/ auxin treated fruit set in spite of over expression of enzymes related to starch (Fig. 5, Additional file 6). These evidences suggest that fruit set induced the availability of glucose and fructose free, not linked in complex polymers. Nevertheless, glucose and fructose levels were lower during parthenocarpic fruit set (Fig. 5), despite the activation of pyruvate kinase or glyceraldehyde-3-phosphate (Additional file 6).

### Auxin, ethylene and gibberellin interaction regulates fruit set in zucchini

The activation of auxin and gibberellin signalling pathways, associated with repression of ethylene signals, trigger fruit set in *Arabidopsis* or tomato, [42], but the role of these hormones is still poorly understood in zucchini fruit set, probably because only few components of these signalling pathways have been indentified and few researches on fruit set has been carried out [5, 43].

Auxins play a critical role in the development of zucchini fruit [42, 44, 45]. RNA-seq showed that genes related to this hormone were the most representative in pollinated fruit and auxin treated fruit. Processes related to auxin signalling were enriched during pollination (Fig. 7) and application of synthetic auxins induced fruit growth in zucchini, causing the development of the ovary into an induced parthenocarpic fruit, suggesting its key role in fruit set [21, 45]. The majority of DEGs related to auxin were up-regulated, and most of them belong to Aux/IAA and PIN families (Fig. 7). Current models suggest that *Aux/IAA* genes encode nuclear proteins which form heterodimers with *ARFs*, and these heterodimers restrain the transcription of the early auxin response genes [46–48]. However, it was found over-expression of *IAA4*, *IAA14* and *IAA16* during fruit set not only in zucchini but also in tomato [22]. This particular regulation of these *IAAs* during fruit set, suggest that a minimum level of *Aux/IAAs* is required in order to create a negative feedback loop in the auxin signal transduction pathway, which enables the plant to fine-tune the strength of the auxin response. Moreover, over-expression of *PIN1*, *PIN6* and *WAT-*1 found in zucchini fruit (Fig. 7), suggested that auxin response is also mediated by auxin polar transport in fruit tissues [42]. This regulation is translated into cell division and into cross-talk with other hormones such as ethylene and gibberellins [31, 42], most representative hormones together auxins during zucchini fruit (Fig. 7). Probably, downregulation of ethylene related genes (Fig. 7, Additional file 7) in zucchini fruit is caused by auxin. Addition of synthetic auxin or pollination has significant effects on the expression of the auxin related genes, regulating negatively ethylene production and signalling during fruit set [43]. *ACO1*, *ACO3*, *ETR2* and EIN3-like genes were downregulated during fruit set (Fig. 7), and this downregulation has also reported previously in zucchini fruit [43], indicating that that ethylene production needs to be prevented for a proper fruit set. This crosstalk between auxin and ethylene has also been reported in tomato [21], displaying that the mRNA levels of several ethylene biosynthesis genes and genes involved in ethylene signalling decreased after pollination and recently, *ETR2* inhibition resulted in earlier fruit set [49].

At the same time, downregulation of ABA biosynthesis related genes (Fig. 7), *NCED1* and *NCED3*, occurred, suggested that auxin response also may imply the attenuation of ABA response [50]. Conversely, cluster analysis of DEGs revealed the strongly over-expression of a key role in gibberellin signalling (Fig. 7), *GA20ox1* and downregulation of genes related with gibberellin catabolism (GID family), indicating that fruit set depends not only on auxin signalling activation but also on induction of gibberellin signalling pathway (Fig. 7). Similar regulation of these genes has been described previously in *Arabidopsis* and tomato during fruit development [8, 42]. In the case of Arabidopsis, *GA20ox1* expression was increased in the ovules, whereas GA2ox gene expression was downregulated. Moreover, in the case of tomato fruit, *GID1B* and GA2ox genes were downregulated in contrast to *GA20ox1* during fruit set, which was strongly up-regulated. However, this regulation does not appear to be independent of auxin, since auxin stimulates gibberellin biosynthesis through the transcriptional activation of *GA20ox1* [8, 42]. These findings suggested that zucchini fruit development depends on the successful activation of the auxin and gibberellin signalling pathways as occurred in tomato.

### Key genes for parthenocarpic fruit set in zucchini

Coordination between auxin, ethylene and gibberellin signalling pathways has proven to be an essential process for fruit set also in zucchini. The role of these hormones is still unknown in parthenocarpic process in zucchini. Differential expression analysis carried out in this assay have revealed a group of genes that showed the same regulation during pollinated/auxin treated and parthenocarpic fruit set (Additional file 7). *ARF18*, *PIN1B*, *PIN8* and *LAX1* were up-regulated like *GA20ox1* and *GASA4* gibberellin related genes This expression profile was similar to found in tomato or *Arabidopsis*, indicating the important role of these genes in parthenocarpy [22, 42]. Remarkably, over-expression of *GA20ox1* gene seems to be crucial for fruit set and parthenocarpy process in zucchini. Moreover, this gene regulated fruit set in coordination of auxin signalling through the transcriptional activation of *ARFs/IAAs* as *ARF18* [42]. The activation of auxin signaling repressed ethylene response in zucchini [43]. This repression was also observed during parthenocarpic fruit set through downregulation of *ACO1, ETR2, ERF11* or *ERF17* (Additional file 7). Gene expression analysis of this subset of genes has represented an important advance in parthenocarpy study in zucchini, and makes possible the introduction of this knowledge in breeding programmes.

## Conclusions

In the present work, transcriptomic changes that take place in zucchini during fruit set have been analysed and compared in two contrasting cultivars for this process, Whitaker, parthenocarpic fruit set, and MUCU-16, non-parthenocarpic fruit set. This study also highlights the crucial role of some pathways including cell cycle, regulation of transcription and carbohydrate metabolism during fruit set in zucchini. Moreover, it was elucidated the important role of hormones during fruit set, establishing the activating role of auxins and gibberellins against the inhibitory role of ethylene. Functional analysis of RNA-Seq data have revealed different candidate genes that could be useful as markers for parthenocarpic selection in the current breeding programs of zucchini.

## Methods

### Plants materials, growth conditions and treatments

Two cultivars of Cucurbita pepo spp. pepo morphotype zucchini were used in this study, the non-parthenocarpic cultivar MUCU-16 (held in COMAV-UPV Genbank, accession BGV004370, https://www.comav.upv.es/index.php/databasesgermplasm/databases/genebank-database) [20], and the parthenocarpic cultivar Whitaker (developed in CORNELL College of Agriculture and Life Science, Geneva, New York, USA) [4]. Those varieties are results of independent traditional breeding process. Seed germination and plant cultivation were performed following standard local practices, with the appropriate permissions to growth plants in a greenhouse of the IFAPA research centre in Almeria (Spain). A mean of 14 h photoperiod, mean daily air temperature of 24 /15 C day/night and relative humidity of 75% were registered in the greenhouse during the experiment.

Female flowers of the above cultivars, at 100 days post transplanting, were protected with paper bags in order to prevent pollen contamination on the day before anthesis. This was followed by three kinds of treatments on each protected flower: non-pollination, pollination and auxin treatment. Paper bags were removed at 1 DPA (day post anthesis) to claim that ovaries were not pollinated in non-pollination treatment. In the case of pollination, paper bags were removed in anthesis and female flowers were self-pollinated by hand early in the morning. In the case of auxin treatment paper bags were removed in anthesis and female flowers were treated with 0.5 ml of 0.8% of synthetic auxin “fruitone” (0.45% 1-naphthalene acetic acid, 1.2% 1-naphthaleneacetamide) for pollinated treatment and auxin treatment, respectively. Ovaries were collected for each treatment from different plants at 2 days post anthesis.

### RNA-Seq analysis

Six fruit samples were collected for RNA-seq analysis, i.e. unpollinated fruit of MUCU-16 (UF MU16), pollinated fruit of MUCU-16 (PF MU16), auxin treated fruit of MUCU-16 (AF MU16), unpollinated fruit of Whitaker (UF WHI), pollinated fruit of Whitaker (PF WHI) and auxin treated fruit of Whitaker (AF WHI). For samples collection, three biological replicates were performed (20 ovaries in each replicate). All collected samples were immediately frozen in liquid nitrogen and stored a − 80 °C until further analysis.

Total RNA was extracted using the RNeasy Plant Mini Kit from (QIAGEN, Germany). 6 libraries for sequencing were constructed for 6 RNA samples, and each sample was the resultant mix of three RNA extraction, one of each sample replication. RNA samples were sent to STABvida (Caparica, Portugal) for RNA-Seq analysis. RNA quality control was performed to evaluate integrity and concentration using Agilen 2200 Tape Station system (Agilent technologies, CA, USA). All the samples were within suitable parameters, RNA amount > 10 μg and RIN > 6.9. Library construction of cDNA molecules was carried out using Illumina TruSeq Stranded mRNA Library Preparation Kit, following manufacter’s instructions. Generate DNA fragments (cDNA libraries) were sequenced in the Illumina HiSeq 2500 platform, using 100 bp paired-end sequencing reads.

### Gene quantification and differential expression analysis

Generated sequence raw data was analyzed using CLC Genomics Worbench 10.0.1. High quality sequencing reads were mapped against the *Curcubita pepo* transcriptome v3 and a processed version of this transcriptome after removal of alternatives sequences, using the following parameters length fraction of 0.80 (at least 80% of the alignment must match the reference sequence before the read is included in the mapping) and similarity fraction of 0.80 (identity should be at least 80% for the read to be included in the mapping). Mapping served to determine the gene expression levels based on the TPM (Transcripts per Million) [51]. Bioconductor package EdgeR was applied to identify differentially expressed genes with multi-factorial statistical analysis tool based on a negative binomial model [52]. A *p-value* could denote its expression difference between two libraries, and false discovery rates (FDRs) were used to determine the threshold of *p-value*. Differentially expressed genes were filtered using standard conditions, fold change (≥ 2 or ≤ − 2) and FDR *p*-Value ≤0.05 [53, 54].

### Gene annotation and gene ontology term enrichment analysis of DEGs

Gene annotation was performed by comparing sequences using algorithm blastn or blastx (cut off e-value of 1e-25) implemented by the program Blast2GO v.4.3.1 [55] with public databases, *Arabidopsis* genome and *Arabidopsis* proteins from TAIR [56], Swissprot [57] and GenBank non redundant nucleotide database (nr) from NCBI [58]. Also, blastn search comparison (cut off e value of 1e-25) was performed with *Cucurbita pepo ssp. ovifera* [59], and *Cucurbita maxima* [60], and with the last versions o*f Cucurbita pepo* genome from Cucurbigene (*https://bioinf.comav.upv.es/downloads/zucchini/genome_v4.1**/*). DEGs were scanned against InterPro database [61] to assign Gene Ontology terms to zucchini genes [62], and GO annotations were mapped to the plant-specific GO slim ontology using Blast2GO with default parameters. Enzyme code mapping was perfomed using GO annotations and pathways The GO term enrichment analysis was conducted using a cut-off *p-value* of 0.05 for significant represented GO Terms and corrections for *p-value* were performed using FDR (false discovery rate). KEGG [63] was used to identified metabolic pathways in DEGs for further understanding genes functions.

### Total sugar measurement

Quantification of sugars in zucchini fruits was conducted using the same samples that have been applied to RNAseq analysis. 12 mg of fruit material were extracted twice with 80% ethanol solution (80% ethanol, 2.5 mM HEPES, pH 7.5) at 95 °C for 30 min, followed by one extraction with 50% ethanol solution (50% ethanol, 2.5 mM HEPES, pH 7.5). The supernatants were combined and used for assaying total soluble sugars, glucose, fructose and sucrose by measuring the difference of absorbance at 340 nm in buffer (75 mM HEPES/KOH pH 7, 2.3 mM ATP, 1 mM NADP and glucose-6-phosphate dehydrogenase) after sequencing adding of hexokinase, phosphoglucose, isomerase, and invertase. For starch determination, the pellets of the ethanol extraction were solubized by heating them to 95 °C in 0.1 M NaOH for 30 min. After acidification with an HCl/sodium-acetate mixture pH 4.9, part of the suspension was digested overnight with amyloglucosidase and α-amylase. The glucose content of the supernant was then used to assess the starch content of the sample by measuring the difference in absorbance at 340 nm after adding hexokinase in the same buffer mentioned above.

## Additional files

Additional file 1: Tables “PF MU16 vs UF MU16” (List of DEGs during pollinated fruit set in MUCU-16 with their respective accession, length, Log_2_ fold change, relative fold change, *p*-value, FDR p-value and Bonferroni), “AFMU16 vs UF MU16 (List of DEGs during auxin treated fruit set in MUCU-16 cultivar with their respective accession, length, Log_2_ fold change, relative fold change, p-value, FDR p-value and Bonferroni), “PF WHT vs UF WHT” (List of DEGs during pollinated fruit set in Whitaker cultivar with their respective accession, length, Log_2_ fold change, relative fold change, p-value, FDR *p*-value and Bonferroni), “AF WHT vs UF WHT (List of DEGs during auxin treated fruit set in Whitaker cultivar with their respective accession, length, Log_2_ fold change, relative fold change, p-value, FDR p-value and Bonferroni), “UF WHT vs UF MU16” (List of DEGs during parthenocarpic fruit set with their respective accession, length, Log_2_ fold change, relative fold change, *p*-value, FDR p-value and Bonferroni). (XLSX 3123 kb) (XLSX 3122 kb)

Additional file 2: Tables “PF MU16 vs UF MU16” (Functional annotation of DEGs during pollinated fruit set in MUCU-16 cultivar based on blast search), “AF MU16 vs UF MU16 (Functional annotation of DEGs during auxin treated fruit set in MUCU-16 cultivar based on blast search), “PF WHT vs UF WHT” (Functional annotation of DEGs during pollinated fruit set in Whitaker cultivar based on blast search), “AF WHT vs UF WHT (Functional annotation of DEGs during auxin treated fruit set in Whitaker cultivar based on blast search), “UF WHT vs UF MU16” (Functional annotation of DEGs during parthenocarpic fruit set based on blast search). (XLSX 6796 kb) (XLSX 6880 kb)

Additional file 3: Tables “PF MU16 vs UF MU16” (Ontology terms of protein products for DEGs during pollinated fruit set in MUCU-16 cultivar), “AF MU16 vs UF MU16 (Ontology terms of protein products for DEGs during auxin treated fruit set in MUCU-16 cultivar), “PF WHT vs UF WHT” (Ontology terms of protein products for DEGs during pollinated fruit set in Whitaker cultivar), “AF WHT vs UF WHT (Ontology terms of protein products for DEGs during auxin treated fruit set in Whitaker cultivar), “UF WHT vs UF MU16” (Ontology terms of protein products for DEGs during parthenocarpic fruit set). (XLSX 2238 kb) (XLSX 2237 kb)

Additional file 4: List of DEGs related to cell division identified by accession, gene annotation, log2 fold change and fold change. (XLSX 20 kb) (XLSX 19 kb)

Additional file 5: Tables “PF MU16 vs UF MU16” (List of DEGs related to TFs during pollinated fruit set in MUCU-16 cultivar identified by accession, gene annotation, log2 fold change and fold change), “AF MU16 vs UF MU16 (List of DEGs related to TFs during auxin treated fruit set in MUCU-16 cultivar by accession, gene annotation, log2 fold change and fold change), “PF WHT vs UF WHT” (List of DEGs related to TFs during pollinated fruit set in Whitaker cultivar by accession, gene annotation, log2 fold change and fold change), “AF WHT vs UF WHT (List of DEGs related to TFs during auxin treated fruit set in Whitaker cultivar by accession, gene annotation, log2 fold change and fold change), “UF WHT vs UF MU16” (List of DEGs related to TFs during parthenocarpic fruit set by accession, gene annotation, log2 fold change and fold change). (XLSX 73 kb) (XLSX 72 kb)

Additional file 6: Tables “PF MU16 vs UF MU16” (List of DEGs related to carbohydrate metabolism during pollinated fruit set in MUCU-16 cultivar identified by accession, gene annotation, log2 fold change, fold change, ontology terms and KEGG pathway), “AF MU16 vs UF MU16 (List of DEGs related to carbohydrate metabolism during auxin treated fruit set in MUCU-16 cultivar by accession, gene annotation, log2 fold change, fold change, ontology terms and KEGG pathway), “PF WHT vs UF WHT” (List of DEGs related to carbohydrate metabolism during pollinated fruit set in Whitaker cultivar by accession, gene annotation, log2 fold change, fold change, ontology terms and KEGG pathway), “AF WHT vs UF WHT (List of DEGs related to carbohydrate metabolism during auxin treated fruit set in Whitaker cultivar by accession, gene annotation, log2 fold change, fold change, ontology terms and KEGG pathway), “UF WHT vs UF MU16” (List of DEGs related to carbohydrate metabolism during parthenocarpic fruit set by accession, gene annotation, log2 fold change, fold change, ontology terms and KEGG pathway). (XLSX 54 kb) (XLSX 53 kb)

Additional file 7: Tables “PF MU16 vs UF MU16” (List of DEGs related to hormones during pollinated fruit set in MUCU-16 cultivar identified by accession, gene annotation, log2 fold change, fold change and hormone pathway), “AF MU16 vs UF MU16 (List of DEGs related to hormones during auxin treated fruit set in MUCU-16 cultivar by accession, gene annotation, log2 fold change, fold change and hormone pathway), “PF WHT vs UF WHT” (List of DEGs related to hormones during pollinated fruit set in Whitaker cultivar by accession, gene annotation, log2 fold change, fold change and hormone pathway), “AF WHT vs UF WHT (List of DEGs related to hormones during auxin treated fruit set in Whitaker cultivar by accession, gene annotation, log2 fold change, fold change and hormone pathway), “UF WHT vs UF MU16” (List of DEGs related to hormones during parthenocarpic fruit set by accession, gene annotation, log2 fold change, fold change and hormone pathway). (XLSX 72 kb) (XLSX 71 kb)

## Abbreviations

ACO: ACC oxidases
AF: Auxin treated fruit
AGL: AGAMOUS LIKE
ARF: Auxin response factor
Aux/IAA: Auxin/indole-3-acetic acid
bHLH: Basic helix-loop-helix protein
BLAST: Basic local alignment search tool
CD-hit: Cluster database at high identity with tolerance
CDK: Cyclin dependet kinase
CYC: Cyclin
DEG: Differentially expressed gene
ERF: Ethylene response factor
EXP: Expansin
GH: Grechen hagen
GO: Gene ontology
HD-Zip: Homeobox-leucine zipper protein
LAX: Auxin influx carriers
MU16: MUCU-16 cultivar
PF: Pollinated fruit
*PIN*: Auxin efflux carriers
RNA-seq: RNA sequencing
*SAUR*: Auxin responsive proteins
TF: Transcription factor
UF: Unpollinated fruit
WHT: Whitaker cultivar
